# Diabetes services and care provision

**Published:** 2016-08-09

**Authors:** Nadia Kareem, Chaudhary Muhamamd Juniad Nazar, Syed Nayer Mahmud, Armughan Ahmed, Muhammad Hammad Akhtar

**Affiliations:** ^1^Department of Public Health, University of Bedfordshire, UK; ^2^Department of Nephrology, Shifa International Hospital, Islamabad, Pakistan; ^3^Department of Nephrology, Pakistan Institute of Medical Sciences, Pakistan

**Keywords:** Diabetic patient, Diabetes services, Diabetes management

**Implication for health policy/practice/research/medical education:**

Diabetes mellitus are rising rapidly in worldwide despite advancing diabetes modalities and services. Many researches are in charge to provide unique healthcare system for diabetic condition management.

Diabetes mellitus are rising rapidly in worldwide despite advancing diabetic modalities and services. This is depicted by a recent study were conducted to improve presented services for diabetic children (1). In this study questionnaire were provided and sent to more than 400 clinicians that took care of diabetic children are less than 16 years old and young diabetic individuals in the United Kingdom. 244 clinicians who took care of 17 thousand diabetic children, completed and sent back questionnaire. Of 244 consultants, around 77% were seen taking a special interest in diabetes mellitus and 90% were dealing with children in a designated diabetic clinic. A specialist nurse was visited 92% of these clinics (more than 40% were untrained to care for children), also a pediatric dietician was visited 64% of these clinics. They found, 88% measured glycated haemoglobin routinely at their clinics, 87% performed retinopathy screening and finally 66% measured microalbuminuria. Non-specialist clinics met significantly fewer of the recommendations of good practice described by Diabetes UK. The survey reveals improvements in services provided for children with diabetes, however serious deficiencies remain. Mentioned services confirm the need for more consultant pediatricians to receive specialist training.

Similarly, a cross sectional study was conducted between April 2008 and January 2009 (2) with to evaluate the perceptions of general practitioners and secondary care health professionals regarding the role of general practitioners in providing preconception care to women with diabetes. Semi-structured interviews were conducted with 22 women having type 1 or type 2 diabetes, belonging to varied ethnic backgrounds who were attended a London teaching hospital or the general practitioners in charge in the hospital catchment area. Seven secondary health professionals associated and eight general practitioners serving the catchment area of the hospital were also parts of the sample of this study. Results of this study indicates, a difference in perceptions of general practitioners and secondary health carers regarding the number of women demanding preconception care.

Likewise, a retrospective cohort study was carried-out to observe the management of diabetes between 2001 and 2007 in the United Kingdom and to assess whether changes in the quality of care reflect existing temporal trends or is a direct result of the implementation of the quality and outcomes framework. Type 1 and 2 diabetic patients were recruited from 147 general practices having continuous data over a 10-year period (from 1 April 1997 to 31 March 2007) (3). The incidence of both type 1 and type 2 diabetes was measured on 31st March every year during the 6 years. Results of the investigation indicate that during 6 years period of the study a significant improvement in diabetes care after the introduction of quality and outcome frameworks was seen. However this improvement does not seem to be a direct result of the quality and outcomes framework. It was found that the introduction of the quality and outcomes framework did not lead to improvement in the management of patients with type 1 diabetes, also not caused a reduction in the number of patients with type 2 diabetes who had hemoglobin A1c (HbA1c) levels greater than 10% (4). They found ,an organized group education programme for individuals with recently diagnosed type 2 diabetes will resulted in better improvements in weight loss, control of diabetes and positive improvements in beliefs about illness (4).

Finally, diabetes mellitus is associated with raised morbidity and premature death from various disease including heart disease, mostly by stroke and myocardial infarction. Hence primary care will led to improved levels of glycaemic control, particularly in patients with type 2 diabetes and a decrement in the mortality.

## Authors’ contribution

All authors contributed to the manuscript equally.

## Conflicts of interest

The authors declared no competing interests.

## Ethical considerations

Ethical issues (including plagiarism, data fabrication, double publication) have been completely observed by authors.

## Funding/Support

None.

## References

[1] Jefferson IG, Swift PG, Skinner TC, Hood GK (2003). Diabetes services in the UK: third national survey confirms continuing deficiencies. Arch Dis Child.

[2] Mortagy I, Kielmann K, Baldeweg SE, Modder J, Pierce MB (2010). Integrating preconception care for women with diabetes into primary care: a qualitative study. Br J Gen Pract.

[3] Calvert M, Shankar A, McManus RJ, Lester H, Freemantle N (2009). Effect of the quality and outcomes framework on diabetes care in the United Kingdom: retrospective cohort study. BMJ.

[4] Davies MJ, Heller S, Skinner TC, Campbell MJ, Carey ME, Cradock S (2008). Effectiveness of the diabetes education and self-management for on-going and newly diagnosed (DESMOND) programme for people with newly diagnosed type 2 diabetes: cluster randomised controlled trial. BMJ.

